# Model-Based Analysis of Costs and Outcomes of Non-Invasive Prenatal Testing for Down’s Syndrome Using Cell Free Fetal DNA in the UK National Health Service

**DOI:** 10.1371/journal.pone.0093559

**Published:** 2014-04-08

**Authors:** Stephen Morris, Saffron Karlsen, Nancy Chung, Melissa Hill, Lyn S. Chitty

**Affiliations:** 1 Department of Applied Health Research, University College London, London, United Kingdom; 2 NHS Fetal Anomaly Screening Programme, University of Exeter, Exeter, United Kingdom; 3 Clinical and Molecular Genetics Unit, UCL Institute of Child Health and Great Ormond Street Hospital for Children NHS Foundation Trust, London, United Kingdom; 4 Fetal Medicine Unit, University College London Hospitals NHS Foundation Trust, London, United Kingdom; Institut Jacques Monod, France

## Abstract

**Background:**

Non-invasive prenatal testing (NIPT) for Down’s syndrome (DS) using cell free fetal DNA in maternal blood has the potential to dramatically alter the way prenatal screening and diagnosis is delivered. Before NIPT can be implemented into routine practice, information is required on its costs and benefits. We investigated the costs and outcomes of NIPT for DS as contingent testing and as first-line testing compared with the current DS screening programme in the UK National Health Service.

**Methods:**

We used a pre-existing model to evaluate the costs and outcomes associated with NIPT compared with the current DS screening programme. The analysis was based on a hypothetical screening population of 10,000 pregnant women. Model inputs were taken from published sources. The main outcome measures were number of DS cases detected, number of procedure-related miscarriages and total cost.

**Results:**

At a screening risk cut-off of 1∶150 NIPT as contingent testing detects slightly fewer DS cases, has fewer procedure-related miscarriages, and costs the same as current DS screening (around UK£280,000) at a cost of £500 per NIPT. As first-line testing NIPT detects more DS cases, has fewer procedure-related miscarriages, and is more expensive than current screening at a cost of £50 per NIPT. When NIPT uptake increases, NIPT detects more DS cases with a small increase in procedure-related miscarriages and costs.

**Conclusions:**

NIPT is currently available in the private sector in the UK at a price of £400-£900. If the NHS cost was at the lower end of this range then at a screening risk cut-off of 1∶150 NIPT as contingent testing would be cost neutral or cost saving compared with current DS screening. As first-line testing NIPT is likely to produce more favourable outcomes but at greater cost. Further research is needed to evaluate NIPT under real world conditions.

## Introduction

In the UK the National Screening Committee (NSC) sets the standards for antenatal screening and recommends that all pregnant women are offered Down’s syndrome (DS) screening. Ideally this is the combined screening test performed between 11 and 14 weeks gestation. In current National Health Service (NHS) practice this has a detection rate of around 85% and a screen postive rate around 2.5% [1]. Women with a risk of 1∶150 or greater of the baby having DS are offered an invasive diagnostic test (chorionic villus sampling (CVS) or amniocentesis), providing definitive diagnosis as to whether or not the baby has DS. If full karyotyping or microarray analysis is performed other chromosomal abnormalities may be detected.

Current invasive diagnostic tests have a risk of miscarriage of 0.5–1% [2]. The discovery of cell free fetal DNA (cffDNA) in maternal blood [3] has led to safer non-invasive approaches to prenatal testing where aneuploidies are detected via a maternal blood test from 10 weeks gestation [4]. Several large-scale validity studies have been conducted to evaluate non-invasive prenatal testing (NIPT) for DS based on next generation sequencing [5]–[12]. Detection rates for DS are typically greater than 99% with a false positive rate of 0.1–1%. NIPT can also detect other aneuploidies including trisomy 18 (99% accurate) and trisomy 13 (up to 90% accurate) [9]–[12]. The small false positive rate for DS means NIPT should be confirmed by invasive testing [13]–[15]. NIPT for DS as well as trisomy 18 and 13 is now offered through commercial providers in several countries including the USA, Germany, Hong Kong and China. It is also available in the private sector in the UK, with prices varying from £400–£900 [16] and samples being sent for testing overseas.

In publicly funded health care systems like the UK NHS, NIPT for DS has the potential to dramatically alter the way prenatal screening and diagnosis is delivered. Before NIPT can be implemented into routine practice, further information is required to identify where it fits in the screening pathway, based on the likely costs and benefits. Several studies have investigated costs and benefits of NIPT for DS using cell free fetal DNA in maternal blood [6], [17]–[21] but there is no evidence that is directly relevant to the UK NHS. Palomaki et al [6] calculated the costs and outcomes of NIPT as contingent testing for DS in the USA. In 100,000 women at high risk for DS, they calculated that invasive testing alone would detect 3,000 DS cases and cost US$100 million with 500 procedure-related losses. NIPT followed by invasive testing in those with positive results would detect 2,958 DS cases at cost of US$3.9 million and 20 procedure-related losses. The authors assumed 100% uptake of NIPT and invasive testing and did not include the cost of NIPT.

Wald and Bestwick [17] investigated the costs and outcomes of a protocol combining DS screening and NIPT, where women at highest risk for DS following screening receive NIPT. Results are presented for different assumptions about the cost of NIPT and the proportion of women eligible for NIPT. The analysis did not include costs and outcomes associated with invasive diagnostic testing.

Garfield and Armstrong [18] assessed the costs and outcomes of NIPT as contingent testing for trisomies 13, 18 and 21 in the USA. In a hypothetical cohort of 100,000 pregnancies they calculated that NIPT as contingent testing reduces the number of procedure-related fetal losses from 60 to 20, increases the number of DS cases detected from 148 to 170, and reduces prenatal testing costs by 1%. They calculated that NIPT as contingent testing is likely to be cost saving at a price of up to US$1,200 per test.

Song et al [19] assessed the cost-effectiveness of NIPT for DS in the USA, comparing first trimester combined screening, integrated screening and NIPT (without conventional screening in women aged 35 years or more, and following a positive screening result in women younger than 35 years). At a cost of US$795, NIPT dominated the other strategies, detecting more DS cases, with fewer procedure-related miscarriages and lower costs. The cost savings were mainly due to reductions in the costs of managing DS during the first five years of life. The authors did not include the medical costs associated with unaffected children during their first five years of life.

Ohno and Caughey [20] analysed the cost-effectiveness of NIPT as contingent testing versus NIPT as a diagnostic test that did not require confirmatory invasive diagnostic testing in the USA. Making assumptions about the quality adjusted life expectancy beyond pregnancy from testing, they concluded that NIPT as contingent testing was cost-effective. The analysis did not compare NIPT versus current practice, so it was not possible to determine if NIPT ought to be adopted.

Cuckle et al [21] investigated factors affecting the cost of avoiding a DS birth using either universal NIPT or NIPT as contingent testing versus conventional screening in the USA. They found that unit costs of NIPT and uptake of NIPT were important factors affecting cost-effectiveness. The high cost of NIPT (US$500-2000 compared with US$150 for the Combined test) meant that universal NIPT was unlikely to be cost-effective.

Our aim was to assess the costs and outcomes of NIPT for DS as contingent testing and as first-line testing compared with the current DS screening programme in the UK NHS. We find that NIPT as contingent testing can produce favourable outcomes at the same cost compared with current DS screening. As first-line testing NIPT is likely to produce more favourable outcomes but at greater cost.

## Materials and Methods

### Ethics Statement

Data used to populate the model were obtained from publicly available sources listed in Table 1. All the data were group averages taken from multiple previously published sources, based on patient data that were de-identified prior to being used in our study. Hence, ethical approval was not required.

**Table 1:** Key model inputs.

Parameter	Value	Source/reference
**Outcomes**		
Maternal age (%)	0.0024–0.0165 by year of maternalage from ≤13 years to ≥50 years	[24]
Prevalence of Down’s syndrome (%)	0.1–2.6 by gestational week and maternal age	[25]
Screening late arrivals (%)	15	[1]
Screening uptake (%)	69	[26]
Screening test performance (DR [%]; FPR [%])		
Combined test with screening riskcut-off of 1 in		
150	85.0; 2.5	[27]
500	94.0; 7.0	
1000	96.0; 12.0	
2000	98.0; 19.0	
Quadruple free β-hCG test	80.5; 4.0	[28]
Weekly spontaneous fetal loss (%)		
DS affected pregnancies	0.5–7.1 by gestational week	[29]
Unaffected pregnancies	0.04–0.07 by maternal age	[30]
NT measurement failures (%)	14–19 by gestational week	[31]
Invasive diagnostic test uptake (%)		
Unaffected pregnancies	80	[32]
DS pregnancies	90	[32]
Invasive diagnostic test performance (DR [%];sample failure rate [%]; procedural miscarriage rate [%])		
CVS/QF-PCR	100; 1.3; 0.5	[33], [30], [33]
Amniocentesis/full karyotyping	100; 0.8; 0.5	[33], [30], [33]
TOP uptake (%)	92.1	[34]
Live birth outcomes (%)		
Vaginal live birth	75.2	[35]
Caesarean live birth	24.8	[35]
**Costs**		
Costs of screening		
Combined test	27	[22]
Quadruple free β-hCG test	35	[22]
Cost invasive diagnostic test (£)		
CVS/QF-PCR	479	[36]
Amniocentesis/full karyotyping	479	[36]
Cost fetal loss (£)		
Spontaneous	511	[36]
Due to CVS or amniocentesis	511	[36]
Cost TOP (£)		
First trimester	697	[36]
Second trimester	882	[36]
Cost live birth outcomes (£)		
Vaginal live birth	1,341	[36]
Caesarean live birth	2,436	[36]
**NIPT**		
Uptake of NIPT (%)		
As contingent testing		
Base case		
Unaffected pregnancies	80	Assumption**
DS pregnancies	90	Assumption**
Alternative scenario	100*	Assumption
Alternative scenario	100*, uptake of screening increase from 69% to 79%	Assumption
As first-line screening		
Base case	69	Assumption***
Alternative scenario	79	Assumption
NIPT performance (DR [%]; FPR [%]; sample failure rate [%]; procedural miscarriage rate [%])	99; 1; 5; 0	Assumption based on Table 2
Cost of NIPT	50, 250, 500, 750	Assumption
Cost of taking blood sample	3	[36]

*For both unaffected and DS affected pregnancies.

**As for invasive diagnostic tests.

***As for DS screening. DS  =  Down’s syndrome; NT  =  nuchal translucency; CVS  =  chorionic villus sampling; QF-PCR  =  Quantitative Fluorescence Polymerase Chain Reaction; TOP  =  termination of pregnancy; DR  =  detection rate; FPR  =  false positive rate

### Including NIPT in the DS Screening Pathway

**Figure 1:**
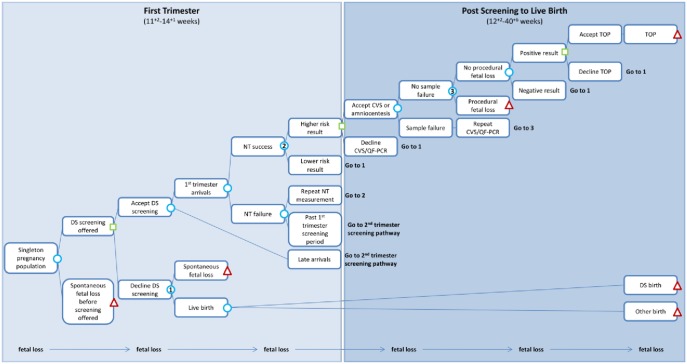
First trimester screening pathway: current DS screening. DS  =  Down’s syndrome; NT  =  nuchal translucency; CVS  =  chorionic villus sampling; QF-PCR  =  Quantitative Fluorescence Polymerase Chain Reaction; TOP  =  termination of pregnancy. See Figure S1 in Supporting Information for the second trimester screening pathway.

**Figure 2:**
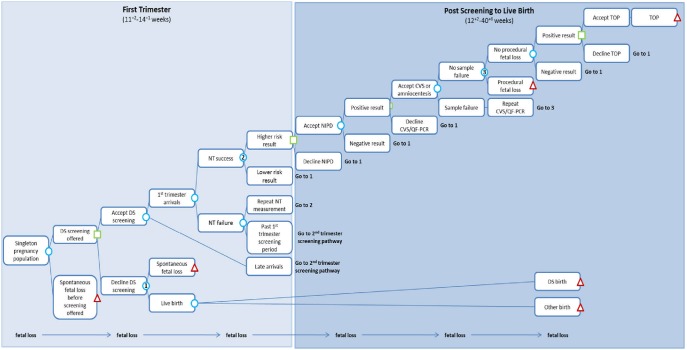
First trimester screening pathway: NIPT as contingent testing. DS  =  Down’s syndrome; NT  =  nuchal translucency; NIPT  =  non-invasive prenatal testing; CVS  =  chorionic villus sampling; QF-PCR  =  Quantitative Fluorescence Polymerase Chain Reaction; TOP  =  termination of pregnancy. See Figure S2 in Supporting Information for the second trimester screening pathway.

**Figure 3:**
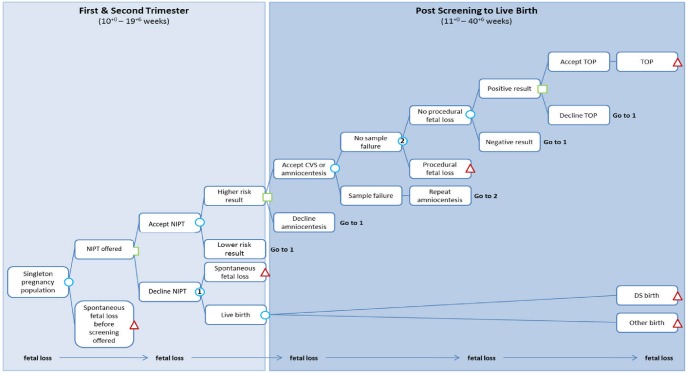
NIPT as first-line testing. DS  =  Down’s syndrome; NT  =  nuchal translucency; NIPT  =  non-invasive prenatal testing; CVS  =  chorionic villus sampling; QF-PCR  =  Quantitative Fluorescence Polymerase Chain Reaction; TOP  =  termination of pregnancy.

We evaluate two approaches to introducing NIPT into the current DS screening pathway. For current DS screening in the UK, the components most relevant for this study are that all pregnant women are offered DS screening and those who are high risk are offered an invasive test (Figure 1, Figure S1 in Supporting Information). The first alternative we consider is with NIPT as contingent testing. In this case, all pregnant women are offered DS screening as before and those with a risk from DS screening above a pre-specified level are offered NIPT rather than an invasive test (Figure 2, Figure S2 in Supporting Information). Invasive testing is then offered to those with abnormal NIPT results. The second alternative is with NIPT as first-line testing, replacing the current DS screening programme (Figure 3). In this case women are not offered DS screening, but are offered NIPT instead and invasive testing is offered to those with abnormal NIPT results. We focused on DS, and did not consider NIPT for trisomies 18 and 13 as DS is the focus of the National Screening Programme.

### Overview of Modelling Approach

The Decision Planning Tool (DPT) is a complex publicly available decision analytic model developed, using Microsoft Excel 1997–2003, by the Peninsula Technology Assessment Group (PenTAG) [22]. It was commissioned by the NHS Fetal Anomaly Screening Programme (FASP), with partial funding from the Knowledge Transfer Partnership (KTP), as a health economic model to support decision-makers and commissioners when assessing the costs and effects of DS screening. The model was developed to include a decision tree structure for six DS screening strategies used within the NHS (double, triple, quadruple, combined, serum integrated and integrated tests) following nationally agreed standards and pathways provided by the NHS FASP. With permission, we amended the DPT to include NIPT as contingent testing and first-line testing. The DPT uses a range of published and publically available national data on the UK costs and estimated prevalence of DS in the UK population as well as the costs of different aspects of DS screening and diagnostic provision. It was specifically designed to assess the costs and benefits of different DS screening tests. For this study, we assumed that all women screened during the first trimester receive the combined test (comprising nuchal translucent measurement combined with analysis of Pregnancy-associated plasma protein-A (PAPP-A) and Free beta-human chorionic gonadotrophin (β-hCG)), and all screened during the second trimester received the quadruple test (Alphafetoprotein (AFP), Free β-hCG or Total hCG, unconjugated oestrial (uE3), and Inhibin-A), since these are recommended tests in the UK [23]. The conclusions do not change when other screening tests are used instead. All stages of the DS screening pathways described in the Figures are accounted for in the adapted DPT model. The perspective was the National DS screening programme in the NHS in the UK. The time horizon over which costs and outcomes are measured is the duration of pregnancy; discounting is therefore unnecessary. As in the DPT, costs are calculated in 2011/12 UK£, inflated from reported values where appropriate.

### Model Inputs

All model inputs, except those for NIPT, for DS screening test performance at different risk cut-offs, and procedural miscarriage rates for invasive diagnostic tests, are as used in the original DPT model and taken from published sources [1], [22], [24]–[36] (Table 1). The screening population is representative of the screening population of England. Maternal age distributions are from the Office for National Statistics’ annual maternity statistics [24]. The underlying prevalence of DS varied by maternal age and gestational ages [25].

Eighty-five per cent of women were offered DS screening during the first trimester using the combined test; 15% were booked after 14 weeks’ gestation and were offered screening in the second trimester with the quadruple test [1]. The uptake rate for both tests was 69% [26].

The detection rate (DR) and false positive rate (FPR) for the combined test were based on different screening risk cut-offs from 1∶150 to 1∶2000 [27]. We used the 1∶150 risk cut-off to evaluate the costs and outcomes associated with current DS screening, and we evaluated NIPT as contingent testing using different screening risk cut-offs from 1∶150 to 1∶2000. The DR and FPR for the quadruple test were 80.5% and 4%, respectively [28].

CVS (amniocentesis) uptake is the proportion of women who accept a first trimester (second trimester) invasive prenatal diagnostic investigation after receiving a high risk screening result. Evidence suggests that for both tests the uptake is 80% for unaffected pregnancies and 90% for DS affected pregnancies [32]. We use these values in the model of current screening and also, in the absence of data, for uptake of invasive diagnostic testing after NIPT. Values for invasive test detection rates and sample failure rates were taken from published figures [30], [33]. The original DPT assumes procedural miscarriage rates of 1% for amniocentesis and 2% for CVS. Based on a recent review [2], which found rates of 0.5–1% for both types of test, we assumed a procedural miscarriage rate for CVS and amniocentesis of 0.5%.

The probability of weekly spontaneous fetal loss for unaffected pregnancies was assumed to be 0.0012 during gestational weeks 10 to 25 and 0.00034 during weeks 26 to 40 [30]. For DS affected pregnancies, it was 0.07067 in weeks 10 to 15 and 0.0051 in weeks 16 to 40 [29]. Termination of pregnancy (TOP) uptake is the proportion of women who accept to terminate their pregnancy after receiving a positive diagnosis for DS and is estimated to be 92.1% [34]. Information on the proportion of women who have a vaginal or caesarean delivery is derived from annual maternity services data [35].

The unit costs of the combined test and the quadruple test were £27 and £35, respectively, including staffing, administration, equipment, overheads, delivery of low risk results via 2^nd^ class mail and high risk results via telephone. The weighted unit cost per CVS/QF-PCR and amniocentesis/full karyotype is estimated to be £479 for each test [36]. The unit cost for spontaneous fetal loss and fetal loss due to an invasive diagnostic procedure are both estimated as £511. The unit cost of TOP was assumed to be £697 in the first trimester and £882 in the second trimester. Unit costs for live birth outcomes were £1,341 for vaginal live birth and £2,436 for caesarean live birth.

A number of studies have reported values for the sensitivity and specificity of NIPT [5–12], with values ranging from 98.6%–100% and 99.7–100%, respectively, and combined values of 99.33% and 99.94%, respectively (Table 2). There were few false negatives, and these occurred in the larger whole genome sequencing studies. We assumed a DR and FPR for NIPT of 99% and 1%, respectively. The sample failure rate for the NIPT was assumed to be 5%. We assumed that when sample failure occurred the cost of another NIPT was incurred; sample failure with NIPT was assumed not to occur more than once in each pregnancy. The procedural fetal loss rate associated with NIPT was 0%. There is limited evidence from the UK to suggest that uptake rate of NIPT in a general DS screening population is higher than current testing [37], a view supported by some small studies of women undergoing NIPT for aneuploidy in the USA [38], but this is currently unproven under real world conditions. For NIPT as contingent screening, we therefore assumed in the base case that uptake of NIPT would be the same as for invasive testing in the current DS screening programme (80% for unaffected pregnancies and 90% for DS affected pregnancies). We also investigated what happened when the NIPT uptake rate increased to 100% and when it increased to 100% with a ten percentage point increase in the uptake of DS screening (69% to 79%). For NIPT as firs- line testing we assumed in the base case that uptake of NIPT would be the same as for DS screening (69%). We also investigated what happened when NIPT uptake increased to 79%. Since NIPT is currently not available via the NHS the actual cost of NIPT is unknown. We therefore used a range of values and present results for costs of NIPT of £50, £250, £500 and £750 per test. In the private sector in the UK, prices for NIPT vary from £400–£900. Figures using a value of £500 are presented, since this represents a threshold value in our results; we do not present results using cost greater than £750 since NIPT is not cost effective above this level; values of £50 and £250 are used to illustrate the cost implications if NIPT costs were to fall substantially beyond current levels in the private sector. We assumed that samples for NIPT were collected at the same time that blood samples for DS screening were collected, and therefore no additional sampling costs were incurred. We also examined an alternative scenario in which women at high risk following DS screening are asked for an additional blood sample for NIPT.

**Table 2:** Sensitivity and specificity of NIPT for Down’s syndrome: results from eight studies.

	Sequencing approach	Test results				Sensitivity		Specificity	
		TP	FN	TN	FP	%	95% CI (%)	%	95% CI (%)
Enrich et al 2011 [5]	Whole genome	39	0	410	1	100	89–100	99.7	98.5–99.9
Palomaki et al 2011 [6]	Whole genome	212	3	1471	3	98.6	98.6–99.5	99.8	99.4–99.9
Bianchi et al 2012 [7]	Whole genome	89	0	404	0	100	95.9–100	100	99.1–100
Ashoor et al 2012 [8]	Targeted	50	0	297	0	100	92.1–100	100	98.7–100
Sparks et al 2012* [9]	Targeted	36	0	123	0	100	90.4–100	100	97.0–100
Norton et al 2012 [10]	Targeted	81	0	2888	1	100	95.5–99.65	99.97	99.8–99.99
Futch et al 2013 [11]	Whole genome	154	2	5515	1	98.72	95.45–99.65	99.98	99.9–100
Liang et al 2013 [12]	Whole genome	40	0	372	0	100	91.24–100	100	98.98–100
**Merged data**		736	5	11601	6	99.33	98.43–99.71	99.94	99.88–99.97

*Calculated using the Fetal-fraction Optimized Risk of Trisomy Evaluation (FORTE) algorithm. TP  =  number of true positive results; FN  =  number of false negative results; TN  =  number of true negative results; FP  =  number of false positive result.

### Outcome Measures

Results are reported for a hypothetical screening population of 10,000 pregnant women who are representative of the screening population of England. The main outcomes are the number of DS cases detected, the number of procedure-related miscarriages and the combined cost of screening, NIPT and invasive diagnostic tests. The cost of screening includes the cost of the screening test (combined test in the first trimester, quadruple test in the second trimester), the cost of repeat nuchal translucency measurements and the cost of delivering the screening test results. We also report the number of women undergoing screening, the number undergoing NIPT, the number with a positive NIPT result, the number having an invasive diagnostic test, the cost of screening, the cost of NIPT, and the cost of invasive diagnostic tests. We report each measure by screening risk cut-off and cost per NIPT test, and separately for different scenarios of NIPT uptake. We also report the costs of each strategy including the costs of pregnancy outcomes.

## Results

In the current DS screening programme using a screening risk cut-off of 1∶150, 6,882 pregnant women would undergo DS screening, 161 would have an invasive diagnostic test, there would be around one procedure-related miscarriage and 13 DS cases detected (Table 3). The total cost would be £279,000 (Table 4). Under base case assumptions concerning NIPT uptake, with NIPT as contingent testing and at a screening risk cut-off of 1∶150, 6,882 women would undergo DS screening, 154 would undergo NIPT, 13 would have a positive NIPT result, 11 would have a diagnostic test, there would be fewer than one procedure-related miscarriage and 11 DS cases would be detected. The total cost would be £213,000-£322,000 depending on the cost of NIPT. At a screening risk cut-off of 1∶150 NIPT as contingent testing results in fewer procedure-related miscarriages (due to fewer invasive diagnostic tests), slightly fewer DS cases being detected (due to the number of women who are high risk according to NIPT but choose not to have an invasive diagnostic test); and, it costs around the same as current DS screening if the cost per NIPT is £500.

**Table 3:** Outcomes of testing strategies in a screening population of 10,000 pregnant women.

Testing strategy	Screening riskcut-off (1 in)	Number undergoing screening	Number undergoing NIPT	Number with apositive NIPT result	Number having an invasivediagnostic test	Number ofprocedure-relatedmiscarriages	Number of DS cases detected
DS screening using the combined test	150s	6,881.66	0		160.59	0.80	13.24
NIPT as contingent testing	150	6,881.66	153.75	13.30	11.48	0.06	11.26
	500	6,881.66	361.43	14.75	12.71	0.06	12.31
	1,000	6,881.66	591.02	15.26	13.13	0.07	12.55
	2,000	6,881.66	912.32	15.85	13.63	0.07	12.78
NIPT as first-line screening		0	6,881.66	28.02	22.03	0.11	16.49

69% uptake of DS screening using the combined test. 80% uptake of NIPT as contingent screening for unaffected pregnancies and 90% for affected pregnancies. 69% uptake of NIPT as first-line screening.

DS  =  Down’s Syndrome; NIPT  =  non-invasive prenatal testing.

**Table 4:** Costs of testing strategies in a screening population of 10,000 pregnant women.

Testing strategy	Screening riskcut-off (1 in)	Cost per NIPT test	(A) Cost of screening (£000s)	(B) Cost ofNIPT (£000s)	(C) Cost of invasive diagnostic tests (£000s)*	(A)+ (B)+ (C) (£000s)
DS screening using the combinedtest	150		200	0	79	279
NIPT as contingent testing	150	£50	200	8	6	213
	150	£250	200	39	6	244
	150	£500	200	78	6	283
	150	£750	200	116	6	322
	500	£50	200	18	6	225
	500	£250	200	91	6	298
	500	£500	200	183	6	389
	500	£750	200	274	6	480
	1,000	£50	200	30	6	237
	1,000	£250	200	149	6	356
	1,000	£500	200	298	6	505
	1,000	£750	200	448	6	655
	2,000	£50	200	46	7	253
	2,000	£250	200	230	7	438
	2,000	£500	200	461	7	668
	2,000	£750	200	691	7	898
NIPT as first-line screening		£50	0	438	11	449
		£250	0	1,642	11	1,825
		£500	0	3,535	11	3,546
		£750	0	5,255	11	5,266

69% uptake of DS screening using the combined test. 80% uptake of NIPT as contingent screening for unaffected pregnancies and 90% for affected.

pregnancies. 69% uptake of NIPT as first-line screening.

*Including procedural miscarriages. DS  =  Down’s syndrome; NIPT  =  non-invasive prenatal testing

If the screening risk cut-off for NIPT was lowered to 1∶500, 1∶1000 or 1∶2000 there is an increase in the number undergoing NIPT, the number with a positive NIPT result, the number having a diagnostic test, the number of procedure-related miscarriages and the number of DS cases detected. Costs increase such that with a screening risk cut-off of 1∶2000 contingent testing with NIPT costs slightly less than current screening if the cost per NIPT is only £50.

With NIPT as first-line screening, 6,882 would undergo NIPT, 28 would have a positive NIPT result, 22 would have an invasive diagnostic test, there would be less than one procedure-related miscarriage and 16 DS cases would be detected. The total cost would be £449,000–£5,266,000 depending on the cost of NIPT. Hence NIPT as first-line screening results in fewer procedure-related miscarriages (due to the lower numbers of invasive diagnostic tests), more DS cases being detected (due to the greater accuracy of NIPT as compared with DS screening as first-line testing), and costs more even than current DS screening if the cost per NIPT is only £50.

When the costs associated with pregnancy outcomes were included to the base case, these were sufficiently large and similar between the different strategies to mask differences in screening and diagnosis costs between DS screening and NIPT as contingent testing (Table S1 in Supporting Information).

Results are presented using the same methodology but with different assumptions about uptake in Supporting Information. In the case of NIPT as contingent testing, compared with the assumptions made in the base analysis in Tables 3 and 4, when NIPT uptake increases, NIPT detects more DS cases with slightly more procedure-related miscarriages and with a small increase in costs (Tables S2–S3 in Supporting Information). If NIPT uptake increases and the provision of NIPT as contingent testing leads to an increase in uptake of DS screening, NIPT produces fewer invasive diagnostic tests, fewer procedure-related miscarriages, and detects more DS cases compared with current DS screening, for the same or a modest increase in costs depending on the screening risk cut-off (Tables S4–S5 in Supporting Information).

The national average unit cost for a phlebotomist to take a blood sample is £3 [34]. If samples for NIPT were collected for high-risk women following DS screening in a separate visit then the cost associated with NIPT increased by a negligible amount. For example, for NIPT as contingent testing with a screening risk cut-off of 1∶150 154 women undergo NIPT and the cost of the extra blood sample is less than £500, making no appreciable difference to the costs in Table 4.

## Discussion

### Main Findings

We analysed the costs and outcomes of NIPT for DS as contingent testing and first-line testing compared with the current DS screening programme. We found that at a screening risk cut-off of 1∶150 NIPT as contingent testing detects slightly fewer DS cases, has fewer procedure-related miscarriages, and costs the same as current DS screening (around UK£280,000) at a cost of £500 per NIPT. NIPT is currently available in the private sector in the UK with prices varying from £400–£900 [16]. If the cost of NIPT in the NHS was at the lower end of this range then at a screening risk cut-off of 1∶150 NIPT as contingent testing would be cost neutral or cost saving compared with current DS screening. When the screening risk cut-off is lowered the cost per NIPT would need to fall considerably for NIPT to be cost neutral compared with current DS screening. As first-line testing NIPT detects more DS cases, has fewer procedure-related miscarriages, and is more expensive than current screening at a cost of £50 per NIPT. When NIPT uptake increases, NIPT detects more DS cases with slightly more procedure-related miscarriages and with a modest increase in costs.

### Strengths and Limitations

A strength of our study is that we utilised a pre-existing validated cost model of DS screening in the NHS, developed to aid Trusts, commissioners and health professionals plan, improve and monitor DS screening.

A limitation is the lack of data about the cost and uptake of NIPT under real world conditions. Our study is designed to inform the implementation of NIPT and since it is not implemented it is unclear what the cost and uptake of NIPT will be. We present results for a range of values. The £400–£900 price range in the private sector includes the cost of samples being sent and tested overseas; costs are likely to fall if testing is undertaken in the UK, and costs of sequencing are decreasing over time. Further research could evaluate the costs and benefits of UK-based testing and the implications of this for the cost-effectiveness of NIPT.

There is limited evidence to suggest that uptake of NIPT in the general pregnant population may be higher than current DS screening [37] but actual values are unknown. We present results assuming NIPT uptake will be the same as for current DS screening and that it will be 100%, and find that our conclusions are not very sensitive to the uptake rates used. We also assumed that uptake of invasive diagnostic testing after a positive NIPT result is the same as after being found high risk from DS screening. Uptake of invasive diagnostic testing after NIPT may actually be higher because NIPT has a higher positive predictive value than DS screening (Song et al [19] assumed that 99% of patients undergo invasive testing after a positive NIPT). This would mean that the difference in DS cases detected between current DS screening and NIPT as contingent testing will fall.

Another limitation is that NIPT can be used to test for trisomy 18, trisomy 13 and some sex chromosome aneuploidies; we have only looked at NIPT for DS. One shortcoming of NIPT is that it does not detect other chromosomal rearrangements, though this is possible with invasive methods. We have not accounted for this on our model, which focuses on DS. Prenatal diagnosis programmes may need to consider the use of invasive tests in pregnancies where the NIPT test is normal, but increased nuchal translucency or other structural abnormalities suggest other chromosomal rearrangements [39], [40]. This requires further evaluation when NIPT is used in clinical practice.

## Conclusions

Our study has two main implications. First, at a screening risk cut-off of 1∶150 and a cost per NIPT of £500 NIPT as contingent testing appears to offer gains over current DS screening in terms of fewer procedure-related miscarriages and at no additional cost. A cost of £500 per NIPT falls within the range that NIPT is currently offered in the private sector in the UK, and so ought to be achievable in the NHS. Second, while it produces better outcomes in terms of DS cases detected and procedure-related fetal losses, NIPT as first-line screening is more expensive than current DS screening, even at a very low cost per test. Hence, NIPT as first-line testing is unlikely to be attractive to NHS commissioners unless the cost of NIPT was to fall dramatically.

The main uncertainties in our analysis are the uptake of NIPT under real world conditions and the cost of NIPT. We have also assumed that the uptake of invasive diagnostic testing after NIPT is the same as after DS screening. The costs and outcomes of NIPT for DS as contingent testing and first-line testing compared with the current DS screening programme ought to be reassessed once the actual uptake rate of NIPT and the cost of NIPT when delivered at scale in the NHS are known.

## Supporting Information

Figure S1 **Second trimester screening pathway: current DS screening.**
(TIF)

Figure S2 **Second trimester screening pathway: NIPT as contingent testing.**
(TIF)

Table S1 **Costs of testing strategies in a screening population of 10,000 pregnant women including costs of pregnancy outcomes.** 69% uptake of DS screening using the combined test. 80% uptake of NIPT as contingent screening for unaffected pregnancies and 90% for affected pregnancies. 69% uptake of NIPT as first line screening.
(DOC)

Table S2 **Outcomes of testing strategies in a screening population of 10,000 pregnant women with alternative assumptions for NIPT uptake.** 69% uptake of DS screening using the combined test. 100% uptake of NIPT as contingent screening. 79% uptake of NIPT as first line screening.
(DOC)

Table S3 **Costs of testing strategies in a screening population of 10,000 pregnant women with alternative assumptions for NIPT uptake.** 69% uptake of DS screening using the combined test. 100% uptake of NIPT as contingent screening. 79% uptake of NIPT as first line screening.
(DOC)

Table S4 **Outcomes of testing strategies in a screening population of 10,000 pregnant women with alternative assumptions for NIPT uptake.** 69% uptake of DS screening using the combined test. 100% uptake of NIPT as contingent screening, plus DS screening uptake increases to 79%. 79% uptake of NIPT as first line screening.
(DOC)

Table S5 **Costs of testing strategies in a screening population of 10,000 pregnant women with alternative assumptions for NIPT uptake.** 69% uptake of DS screening using the combined test. 100% uptake of NIPT as contingent screening, plus DS screening uptake increases to 79%. 79% uptake of NIPT as first line screening.
(DOC)

Table S6 **CHEERS statement.**
(DOC)
