# Supplementary figures and images for: Model-Based Analysis of Costs and Outcomes of Non-Invasive Prenatal Testing for Down’s Syndrome Using Cell Free Fetal DNA in the UK National Health Service
Source: PLoS One. 2014 Apr 8;9(4):e93559. doi: 10.1371/journal.pone.0093559 (PMC3979704; doi:10.1371/journal.pone.0093559)

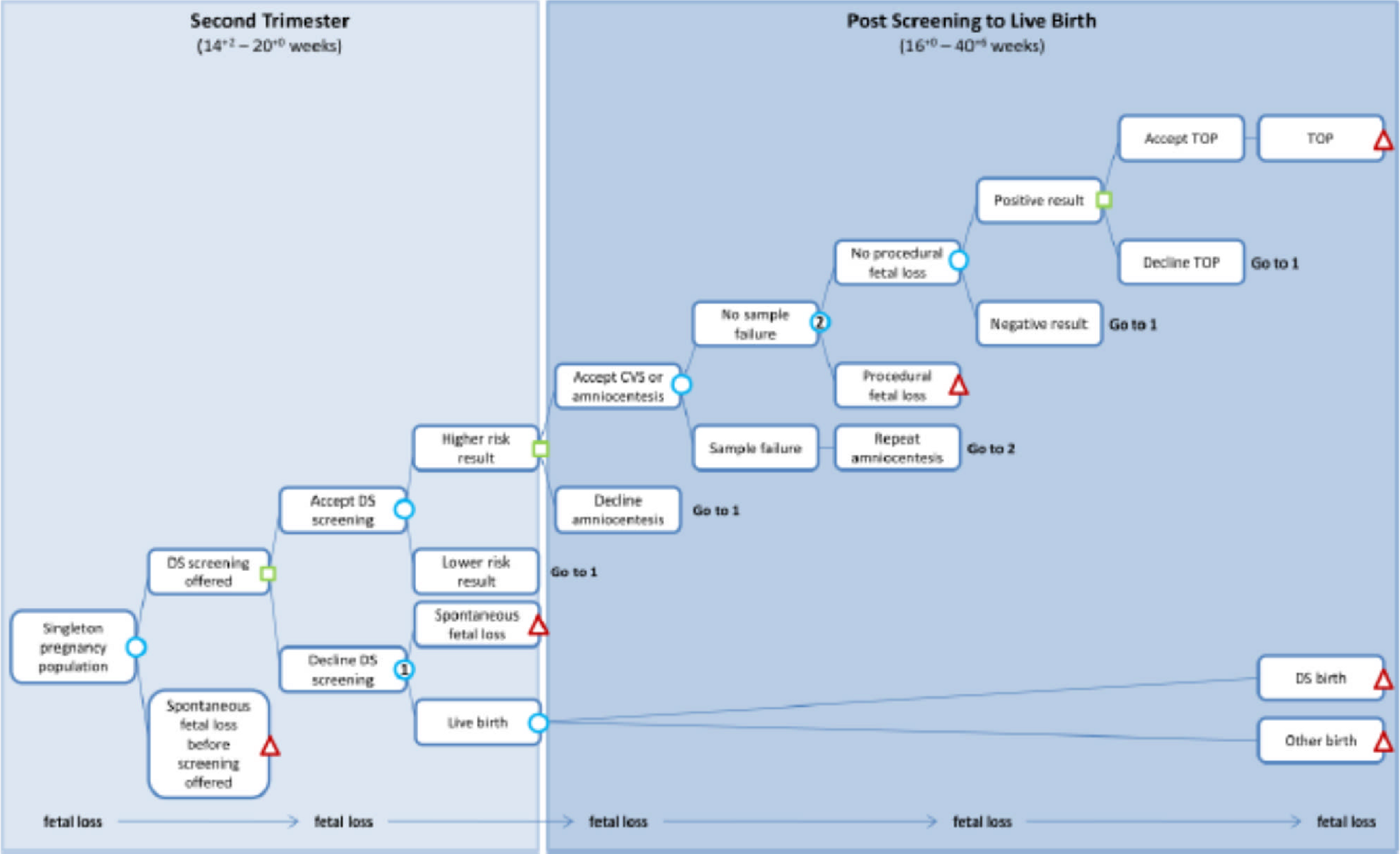

Supplement: Figure S1 — Second trimester screening pathway: current DS screening. (TIF) [file pone.0093559.s001.tif]

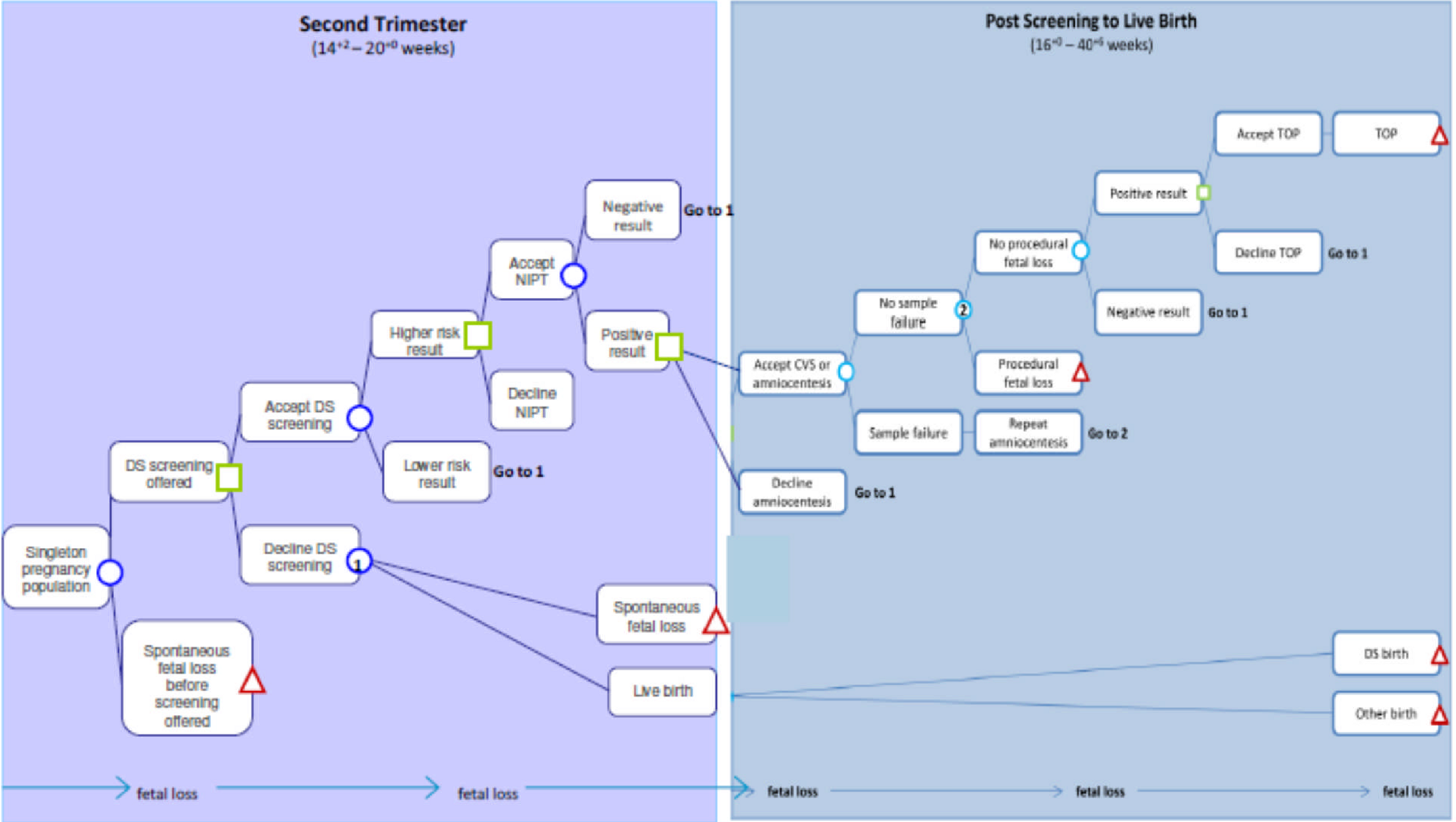

Supplement: Figure S2 — Second trimester screening pathway: NIPT as contingent testing. (TIF) [file pone.0093559.s002.tif]
