# Supplementary material for: Model-Based Analysis of Costs and Outcomes of Non-Invasive Prenatal Testing for Down’s Syndrome Using Cell Free Fetal DNA in the UK National Health Service
Source: PLoS One. 2014 Apr 8;9(4):e93559. doi: 10.1371/journal.pone.0093559 (PMC3979704; doi:10.1371/journal.pone.0093559)
Supplement: Table S1 — Costs of testing strategies in a screening population of 10,000 pregnant women including costs of pregnancy outcomes. 69% uptake of DS screening using the combined test. 80% uptake of NIPT as contingent screening for unaffected pregnancies and 90% for affected pregnancies. 69% uptake of NIPT as first line screening. (DOC) [file pone.0093559.s003.doc]

**Table S1. Costs of testing strategies in a screening population of 10,000 pregnant women including costs of pregnancy outcomes.**

69% uptake of DS screening using the combined test. 80% uptake of NIPT as contingent screening for unaffected pregnancies and 90% for affected pregnancies. 69% uptake of NIPT as first line screening.

| **Testing strategy** | **Screening**  **risk cut-off**  **(1 in)** | **Cost per**  **NIPT test** | **(A)**  **Cost of**  **screening**  **(£000s)** | **(B)**  **Cost of NIPT**  **(£000s)** | **(C)**  **Cost of invasive**  **diagnostic tests**  **(£000s)*** | **(D)**  **Cost of pregnancy outcomes (£000s)**** | **(A)+(B)+(C)**  **(£000s)** | **(A)+(B)+(C)+(D)**  **(£000s)** |
| --- | --- | --- | --- | --- | --- | --- | --- | --- |
| DS screening using the combined test | 150 |  | 200 | 0 | 79 | 15,851 | 279 | 16,130 |
| NIPT as contingent testing | 150 | £50 | 200 | 8 | 6 | 15,854 | 213 | 16,067 |
|  | 150 | £250 | 200 | 39 | 6 | 15,854 | 244 | 16,098 |
|  | 150 | £500 | 200 | 78 | 6 | 15,854 | 283 | 16,137 |
|  | 150 | £750 | 200 | 116 | 6 | 15,854 | 322 | 16,176 |
|  |  |  |  |  |  |  |  |  |
|  | 500 | £50 | 200 | 18 | 6 | 15,853 | 225 | 16,078 |
|  | 500 | £250 | 200 | 91 | 6 | 15,853 | 298 | 16,151 |
|  | 500 | £500 | 200 | 183 | 6 | 15,853 | 389 | 16,242 |
|  | 500 | £750 | 200 | 274 | 6 | 15,853 | 480 | 16,333 |
|  |  |  |  |  |  |  |  |  |
|  | 1,000 | £50 | 200 | 30 | 6 | 15,853 | 237 | 16,090 |
|  | 1,000 | £250 | 200 | 149 | 6 | 15,853 | 356 | 16,209 |
|  | 1,000 | £500 | 200 | 298 | 6 | 15,853 | 505 | 16,358 |
|  | 1,000 | £750 | 200 | 448 | 6 | 15,853 | 655 | 16,508 |
|  |  |  |  |  |  |  |  |  |
|  | 2,000 | £50 | 200 | 46 | 7 | 15,853 | 253 | 16,106 |
|  | 2,000 | £250 | 200 | 230 | 7 | 15,853 | 438 | 16,291 |
|  | 2,000 | £500 | 200 | 461 | 7 | 15,853 | 668 | 16,521 |
|  | 2,000 | £750 | 200 | 691 | 7 | 15,853 | 898 | 16,751 |
| NIPT as first line screening |  | £50 | 0 | 438 | 11 | 15,849 | 449 | 16,298 |
|  |  | £250 | 0 | 1,642 | 11 | 15,849 | 1,825 | 17,674 |
|  |  | £500 | 0 | 3,535 | 11 | 15,849 | 3,546 | 19,395 |
|  |  | £750 | 0 | 5,255 | 11 | 15,849 | 5,266 | 21,115 |

* Including procedural miscarriages. ** TOP, spontaneous fetal loss and live births. DS = Down’s syndrome; NIPT = non-invasive prenatal testing; TOP = termination of pregnancy.
