# Supplementary material for: Model-Based Analysis of Costs and Outcomes of Non-Invasive Prenatal Testing for Down’s Syndrome Using Cell Free Fetal DNA in the UK National Health Service
Source: PLoS One. 2014 Apr 8;9(4):e93559. doi: 10.1371/journal.pone.0093559 (PMC3979704; doi:10.1371/journal.pone.0093559)
Supplement: Table S2 — Outcomes of testing strategies in a screening population of 10,000 pregnant women with alternative assumptions for NIPT uptake. 69% uptake of DS screening using the combined test. 100% uptake of NIPT as contingent screening. 79% uptake of NIPT as first line screening. (DOC) [file pone.0093559.s004.doc]

**Table S2. Outcomes of testing strategies in a screening population of 10,000 pregnant women with alternative assumptions for NIPT uptake**

69% uptake of DS screening using the combined test. 100% uptake of NIPT as contingent screening. 79% uptake of NIPT as first line screening.

| **Testing strategy** | **Screening risk cut-off**  **(1 in)** | **Number undergoing screening** | **Number undergoing NIPT** | **Number with a positive NIPT result** | **Number having an invasive diagnostic test** | **Number of procedure-related miscarriages** | **Number of DS cases detected** |
| --- | --- | --- | --- | --- | --- | --- | --- |
| DS screening using the combined test | 150 | 6,881.66 | 0 |  | 160.59 | 0.80 | 13.24 |
| NIPT as contingent testing | 150 | 6,881.66 | 190.26 | 14.81 | 12.78 | 0.06 | 12.51 |
|  | 500 | 6,881.66 | 449.68 | 16.44 | 14.17 | 0.07 | 13.69 |
|  | 1,000 | 6,881.66 | 736.62 | 17.04 | 14.66 | 0.07 | 13.95 |
|  | 2,000 | 6,881.66 | 1138.21 | 17.75 | 15.25 | 0.08 | 14.21 |
| NIPT as first line screening |  | 0 | 7,879.34 | 32.08 | 25.22 | 0.13 | 18.88 |

DS = Down’s syndrome; NIPT = non-invasive prenatal testing.
