# Supplementary material for: Model-Based Analysis of Costs and Outcomes of Non-Invasive Prenatal Testing for Down’s Syndrome Using Cell Free Fetal DNA in the UK National Health Service
Source: PLoS One. 2014 Apr 8;9(4):e93559. doi: 10.1371/journal.pone.0093559 (PMC3979704; doi:10.1371/journal.pone.0093559)
Supplement: Table S3 — Costs of testing strategies in a screening population of 10,000 pregnant women with alternative assumptions for NIPT uptake. 69% uptake of DS screening using the combined test. 100% uptake of NIPT as contingent screening. 79% uptake of NIPT as first line screening. (DOC) [file pone.0093559.s005.doc]

**Table S3. Costs of testing strategies in a screening population of 10,000 pregnant women** **with alternative assumptions for NIPT uptake**

69% uptake of DS screening using the combined test. 100% uptake of NIPT as contingent screening. 79% uptake of NIPT as first line screening.

| **Testing strategy** | **Screening**  **risk cut-off**  **(1 in)** | **Cost per**  **NIPT test** | **(A)**  **Cost of**  **screening**  **(£000s)** | **(B)**  **Cost of NIPT**  **(£000s)** | **(C)**  **Cost of invasive**  **diagnostic tests**  **(£000s)*** | **(D)**  **Cost of**  **pregnancy**  **outcomes**  **(£000s)**** | **(A)+(B)+(C)**  **(£000s)** | **(A)+(B)+(C)+(D)**  **(£000s)** |
| --- | --- | --- | --- | --- | --- | --- | --- | --- |
| DS screening using the combined test | 150 |  | 200 | 0 | 79 | 15,851 | 279 | 16,130 |
| NIPT as contingent testing | 150 | £50 | 200 | 10 | 6 | 15,852 | 216 | 16,068 |
|  | 150 | £250 | 200 | 48 | 6 | 15,852 | 254 | 16,106 |
|  | 150 | £500 | 200 | 96 | 6 | 15,852 | 302 | 16,154 |
|  | 150 | £750 | 200 | 144 | 6 | 15,852 | 350 | 16,202 |
|  |  |  |  |  |  |  |  |  |
|  | 500 | £50 | 200 | 23 | 7 | 15,851 | 230 | 16,081 |
|  | 500 | £250 | 200 | 114 | 7 | 15,851 | 321 | 16,172 |
|  | 500 | £500 | 200 | 227 | 7 | 15,851 | 434 | 16,286 |
|  | 500 | £750 | 200 | 341 | 7 | 15,851 | 548 | 16,399 |
|  |  |  |  |  |  |  |  |  |
|  | 1,000 | £50 | 200 | 37 | 7 | 15,851 | 245 | 16,096 |
|  | 1,000 | £250 | 200 | 186 | 7 | 15,851 | 394 | 16,245 |
|  | 1,000 | £500 | 200 | 372 | 7 | 15,851 | 580 | 16,431 |
|  | 1,000 | £750 | 200 | 558 | 7 | 15,851 | 766 | 16,617 |
|  |  |  |  |  |  |  |  |  |
|  | 2,000 | £50 | 200 | 57 | 7 | 15,851 | 266 | 16,117 |
|  | 2,000 | £250 | 200 | 287 | 7 | 15,851 | 496 | 16,347 |
|  | 2,000 | £500 | 200 | 575 | 7 | 15,851 | 783 | 16,634 |
|  | 2,000 | £750 | 200 | 862 | 7 | 15,851 | 1,070 | 16,921 |
| NIPT as first line screening |  | £50 | 0 | 501 | 12 | 15,848 | 514 | 16,362 |
|  |  | £250 | 0 | 2,077 | 12 | 15,848 | 2,090 | 17,937 |
|  |  | £500 | 0 | 4,047 | 12 | 15,848 | 4,060 | 19,907 |
|  |  | £750 | 0 | 6,017 | 12 | 15,848 | 6,029 | 21,877 |

* Including procedural miscarriages. ** TOP, spontaneous fetal loss and live births. DS = Down’s syndrome; NIPT = non-invasive prenatal testing; TOP = termination of pregnancy.
