# Supplementary material for: Model-Based Analysis of Costs and Outcomes of Non-Invasive Prenatal Testing for Down’s Syndrome Using Cell Free Fetal DNA in the UK National Health Service
Source: PLoS One. 2014 Apr 8;9(4):e93559. doi: 10.1371/journal.pone.0093559 (PMC3979704; doi:10.1371/journal.pone.0093559)
Supplement: Table S5 — Costs of testing strategies in a screening population of 10,000 pregnant women with alternative assumptions for NIPT uptake. 69% uptake of DS screening using the combined test. 100% uptake of NIPT as contingent screening, plus DS screening uptake increases to 79%. 79% uptake of NIPT as first line screening. (DOC) [file pone.0093559.s007.doc]

**Table S5. Costs of testing strategies in a screening population of 10,000 pregnant women with alternative assumptions for NIPT uptake**

69% uptake of DS screening using the combined test. 100% uptake of NIPT as contingent screening, plus DS screening uptake increases to 79%. 79% uptake of NIPT as first line screening.

| **Testing strategy** | **Screening**  **risk cut-off**  **(1 in)** | **Cost per**  **NIPT test** | **(A)**  **Cost of**  **screening**  **(£000s)** | **(B)**  **Cost of NIPT**  **(£000s)** | **(C)**  **Cost of invasive**  **diagnostic tests**  **(£000s)*** | **(D)**  **Cost of**  **pregnancy**  **outcomes**  **(£000s)**** | **(A)+(B)+(C)**  **(£000s)** | **(A)+(B)+(C)+(D)**  **(£000s)** |
| --- | --- | --- | --- | --- | --- | --- | --- | --- |
| DS screening using the combined test | 150 |  | 200 | 0 | 79 | 15,851 | 279 | 16,130 |
| NIPT as contingent testing | 150 | £50 | 229 | 11 | 7 | 15,851 | 247 | 16,098 |
|  | 150 | £250 | 229 | 55 | 7 | 15,851 | 291 | 16,142 |
|  | 150 | £500 | 229 | 110 | 7 | 15,851 | 345 | 16,196 |
|  | 150 | £750 | 229 | 165 | 7 | 15,851 | 400 | 16,251 |
|  |  |  |  |  |  |  |  |  |
|  | 500 | £50 | 229 | 26 | 8 | 15,850 | 263 | 16,113 |
|  | 500 | £250 | 229 | 130 | 8 | 15,850 | 367 | 16,217 |
|  | 500 | £500 | 229 | 260 | 8 | 15,850 | 496 | 16,347 |
|  | 500 | £750 | 229 | 390 | 8 | 15,850 | 626 | 16,476 |
|  |  |  |  |  |  |  |  |  |
|  | 1,000 | £50 | 229 | 43 | 8 | 15,850 | 280 | 16,130 |
|  | 1,000 | £250 | 229 | 213 | 8 | 15,850 | 450 | 16,300 |
|  | 1,000 | £500 | 229 | 426 | 8 | 15,850 | 663 | 16,513 |
|  | 1,000 | £750 | 229 | 638 | 8 | 15,850 | 876 | 16,726 |
|  |  |  |  |  |  |  |  |  |
|  | 2,000 | £50 | 229 | 66 | 9 | 15,850 | 304 | 16,153 |
|  | 2,000 | £250 | 229 | 329 | 9 | 15,850 | 567 | 16,416 |
|  | 2,000 | £500 | 229 | 658 | 9 | 15,850 | 896 | 16,745 |
|  | 2,000 | £750 | 229 | 987 | 9 | 15,850 | 1,225 | 17,074 |
| NIPT as first line screening |  | £50 | 0 | 501 | 12 | 15,848 | 514 | 16,362 |
|  |  | £250 | 0 | 2,077 | 12 | 15,848 | 2,090 | 17,937 |
|  |  | £500 | 0 | 4,047 | 12 | 15,848 | 4,060 | 19,907 |
|  |  | £750 | 0 | 6,017 | 12 | 15,848 | 6,029 | 21,877 |

* Including procedural miscarriages. ** TOP, spontaneous fetal loss and live births. DS = Down’s syndrome; NIPT = non-invasive prenatal testing; TOP = termination of pregnancy.
