# Supplementary material for: Model-Based Analysis of Costs and Outcomes of Non-Invasive Prenatal Testing for Down’s Syndrome Using Cell Free Fetal DNA in the UK National Health Service
Source: PLoS One. 2014 Apr 8;9(4):e93559. doi: 10.1371/journal.pone.0093559 (PMC3979704; doi:10.1371/journal.pone.0093559)
Supplement: Table S6 — CHEERS statement. (DOC) [file pone.0093559.s008.doc]

**Table S6. CHEERS Statement**

Husereau D, Drummond M, Petrou S, Carswell C, Moher D, Greenberg D, et al on behalf of the CHEERS Task Force (2013) Consolidated Health Economic Evaluation Reporting Standards (CHEERS) statement. Br Med J 346: f1049.

| **Title and abstract** | | | |
| --- | --- | --- | --- |
| Title | 1 | We identify the study as an economic analysis based on the terms “costs” and “outcomes” in the title, plus we describe the intervention being evaluated (non-invasive prenatal testing for Down’s syndrome using cell free fetal DNA). |  |
| Abstract | 2 | A structured summary is provided. |  |
| **Introduction** | | | |
| Background and objectives | 3 | The broader context for the study is clearly described. |  |
|  |  | The research question, and its rationale, is discussed. |  |
| **Methods** | | | |
| Target population and subgroups | 4 | The population is described in the ‘Model inputs’ sub-section. |  |
| Setting and location | 5 | The study setting (the National DS screening programme in the UK) is described in the ‘Introduction’. |  |
| Study perspective | 6 | We explain that the perspective was the National DS screening programme in the NHS in the UK (see ‘Overview of modelling approach’ sub-section). |  |
| Comparators | 7 | The DS screening strategies being compared are described in detail in the ‘Including NIPT in the DS screening pathway’ sub-section. |  |
| Time horizon | 8 | The time horizon is stated in the ‘Overview of modelling approach’ sub-section. |  |
| Discount rate | 9 | We explain that discounting is unnecessary due to time horizon taken in the ‘Overview of modelling approach’ sub-section. |  |
| Choice of health outcomes | 10 | The outcome measures are described in the ‘Outcome measures’ sub-section. |  |
| Measurement of effectiveness | 11b | We explain how the measures of effectiveness are generated from a pre-existing model in the ‘Overview of modelling approach’ sub-section. |  |
|  |  |  |  |
| Measurement and valuation of preference based outcomes | 12 | Not applicable. |  |
| Estimating resources and costs | 13b | Details of the resources used and unit costs are described in the ‘Model inputs’ sub-section. |  |
|  |  |  |  |
| Currency, price date, and conversion | 14 | We explain that costs are calculated in 2012/13 UK£ in the ‘Overview of modelling approach’ sub-section. |  |
| Choice of model | 15 | We justify our choice of model, and describe it in detail (including figures) in the ‘Including NIPT in the DS screening pathway’ and the ‘Overview of modelling approach’ sub-sections. |  |
| Assumptions | 16 | The assumptions underpinning the model are described in the ‘Including NIPT in the DS screening pathway’, the ‘Overview of modelling approach’ and the ‘Model inputs’ sub-sections. |  |
| Analytical methods | 17 | The analytical methods are described in the ‘Including NIPT in the DS screening pathway’, the ‘Overview of modelling approach’ and the ‘Model inputs’ sub-sections. |  |
| **Results** | | | |
| Study parameters | 18 | The main model parameters are in Table 1. |  |
| Incremental costs and outcomes | 19 | The costs and outcomes in the base case are reported in Tables 3 and 4. Incremental cost-effectiveness ratios are not reported. |  |
| Characterising uncertainty | 20b | Evaluation of uncertainty is presented in Tables 3 and 4, and in the Supporting Information. We focus on the role of uncertainty with regards to DS screening cut-offs, NIPT costs, and NIPT uptake. |  |
|  |  |  |  |
| Characterising heterogeneity | 21 | Not applicable. |  |
| **Discussion** | | | |
| Study findings, limitations, generalisability, and current knowledge | 22 | Study findings are summarised in the ‘Main findings’ sub-section. Limitations are described in the ‘Strengths and limitations’ sub-section. How the findings fit with current knowledge is described in the ‘Comparison with other studies’ sub-section. |  |
| **Other** | | | |
| Source of funding | 23 | The funding source and the role of the funder are described. |  |
| Conflicts of interest | 24 | Competing interests are described. |  |
